# Pediatric Palliative Care Initiative in Cambodia

**DOI:** 10.3389/fpubh.2017.00185

**Published:** 2017-07-28

**Authors:** Mahmut Yaşar Çeliker, Yos Pagnarith, Kazumi Akao, Dim Sophearin, Sokchea Sorn

**Affiliations:** ^1^Pediatric Hematology/Oncology and Hospice and Palliative Care, Maimonides Infants’ and Children’s Hospital, Brooklyn, NY, United States; ^2^Pediatric Intensive Care, Angkor Hospital for Children, Siem Reap, Cambodia; ^3^Friends Without A Border, Lao Friends Hospital for Children, Luang Prabang, Laos; ^4^Angkor Hospital for Children, Siem Reap, Cambodia

**Keywords:** palliative care, hospice, Cambodia, children, LIC, HVO

## Abstract

Cancer care with curative intent remains difficult to manage in many resource-limited settings such as Cambodia. Cambodia has a small workforce with limited financial and health-care resources resulting in delayed diagnoses and availability of limited therapeutic tools. Thus, palliative care becomes the primary form of care in most cases. Although palliative care is becoming an integral part of medical care in developed countries, this concept remains poorly understood and utilized in developing countries. Angkor Hospital for Children serves a relatively large pediatric population in northern Cambodia. According to the modern definition of palliative care, approximately two-thirds of the patients admitted to the hospital were deemed candidates to receive palliative care. In an effort to develop a pediatric palliative care team utilizing existing resources and intensive training, our focus group recruited already existing teams with different health-care expertise and other motivated members of the hospital. During this process, we have also formed a palliative care training team of local experts to maintain ongoing palliative care education. Feedback from patients and health-care providers confirmed the effectiveness of these efforts. In conclusion, palliative and sustainable care was offered effectively in a resource-limited setting with adequately trained and motivated local providers. In this article, the steps and systems used to overcome challenges in Cambodia are summarized in the hope that our experience urges governmental and non-governmental agencies to support similar initiatives.

## Introduction

Curative cancer care remains an enormous challenge for developing countries (1, 2). Eighty percent of children with cancer live in countries with limited resources, where more than 90% of childhood cancer deaths occur (3). Even in cases where cure is possible, delayed diagnosis and limited access to care make it virtually impossible to attain it in many developing countries. As such, palliative care frequently becomes the only option for these children.

World Health Organization (WHO) made prevention and control of non-communicable diseases such as cancer priority with the launch of Global Action Plan in 2013 (2, 4). The WHO Global Action Plan includes efforts to improve palliative care aimed at achieving relief of suffering in over 90% of cancer patients. Although palliative care programs have been successfully implemented in a handful of resource-limited countries the concept of palliative care is still in its early stages in many others (5–8). The significant progress made in palliative care for children living with HIV due to a global effort to control the disease did not extend successfully to other life-threatening illnesses; however, it may provide a model for palliative care for children living with cancer (7, 9–11).

## Background and Rationale

Cambodia has made a significant progress in reducing its childhood mortality rate during recent decades (12). This was accomplished by widespread application of preventive measures and improved access to medical care. However, oncological care remains in its early stages. Although cancer care is available in a small number of governmental and private health centers in Cambodia, it is accessible only to a small portion of the population with adequate socioeconomic resources. Care for cancer and other non-communicable chronic and debilitating diseases remains a significant challenge in the general population, especially in pediatrics. As a result, palliative care becomes the primary mechanism of care for many of these patients.

Angkor Hospital for Children (AHC) is an acute care center and offers care to thousands of children with life-threatening illnesses. At any given time, an estimated 60% of the children admitted to the AHC suffer from life-threatening illnesses and could benefit from palliative care in addition to care targeting the disease. At the time of this study, cancer was not considered a curable disease in this hospital’s setting and children with cancer were only offered supportive care when necessary. More recently, with the help of global volunteer efforts, curative cancer care is offered to children with certain cancer diagnoses at the AHC.

The study took place during April and May of 2012 when Mahmut Yaşar Çeliker, the principle author volunteered in AHC with the American Society of Hematology-Health Volunteers Overseas (http://HVOUSA.org). In this report, we will present our efforts to utilize all available resources to form an effective pediatric palliative care service in one of the largest children’s hospitals serving children in Cambodia.

For the duration of our study, palliative care was offered as symptom management by the respective specialists and not as a team effort for children with cancer at AHC. As ongoing educational and material support through twinning and other mechanisms are underway to improve cancer care for children in Cambodia, we have targeted to offer a more effective palliative care to these children. Our initial goal was to provide a model for palliative care to those deemed incurable and to later expand it to all children living with life-threatening illnesses.

## Palliative Care Initiative

### Settings

#### Cambodia Demographics

Population census carried out in 2008 shows that Cambodia’s population is 13,395,682, 45% of which are under the age of 20 (13). Seventy-nine percent of the population lives in rural areas. Per capita GDP is 1,158.7 USD which is 2% of the per capita GDP for the USA (14). An estimated 31% of the population lives below the poverty line for Cambodia standards. Approximately 35% of the children less than 5 years of age are malnourished (i.e., height and weight below 2 SD of the average) (12). Under-five mortality rate in 2011 was 29 per 1,000 live births which are down from 108 in 2,000 (12).

#### AHC Resources

Angkor Hospital for Children is a non-profit pediatric teaching hospital in Siem Reap and offers care for all children. It is equipped with a 42-bed inpatient unit, a 10-bed low acuity unit, and an approximately 10-bed intensive care unit. Basic laboratory support consists of a blood bank that offers whole blood and packed RBCs, and occasionally apheresis platelets; a hematology laboratory that offers hemogram and basic coagulation studies; a chemistry laboratory that offers basic metabolic screen and limited number of more specialized chemistry tests; and a microbiology laboratory. Imaging studies offered at the hospital are limited to X-ray and ultrasonography. Patients requiring CT scan or MRI are sent to other area hospitals where these tools are available for which AHC is charged a fee per use. Patients are charged a nominal fee for the services provided in AHC but all patients regardless of ability to pay are offered care. Hospital pharmacy is equipped with adequate medication for inpatient and outpatient services offering various antibiotics and other medications to serve the community effectively. However, narcotic analgesics are limited to morphine, intravenous fentanyl, meperidine, paracetamol-codeine, and tramadol. Continuing education through didactic teaching sessions by the local faculty as well as volunteer faculty from abroad contribute significantly to the education of health-care providers.

#### Population and Health-care Statistics for AHC

Angkor Hospital for Children serves approximately 1 million people from the province of Siem Reap and northern Cambodia provinces. Malnutrition prevalence in the province of Siem Reap is much higher than that of country average. Approximately half of the children younger than 5 years of age are considered malnourished (i.e., 50% below 2 SD and 20% below 3 SD for height or weight; National Institutes of Statistics data) (12).

On an average, 500 patients are offered outpatient and emergency care daily, which translates to approximately 180,000 patients annually. The top indications for admission at AHC were infections, i.e., respiratory infections, hemorrhagic Dengue fever, gastroenteritis, septicemia, malaria, HIV/AIDS, and non-infectious conditions, i.e., malnutrition (15).

#### Resource Assessment

During the first week of the study time period, a child with fever and bone pain presumed to have osteomyelitis was diagnosed of acute lymphoblastic leukemia (ALL) with bone marrow aspiration. Since there is no hospital in Siem Reap with the resources necessary to offer curative therapy, options were outlined as: (1) sending the patient to Phnom Penh where two hospitals are able to offer chemotherapy as reported by the local hematologist or (2) sending the child home on steroids and attending the child’s needs as he presents to the hospital with complications. Since many people of Cambodia live on 2 USD a day and the poverty rate is significantly higher in Siem Reap than the country’s average, a move to Phnom Penh is financially prohibitive to almost all patients including this child. During the time of this case, patients with leukemia were sent home on steroids and were cared for when they arrived to the emergency room with severe anemia or bleeding. This highlighted the need for better palliative care for these children.

We have also identified children with non-oncological problems who could benefit from better palliative care. For example, patients with thalassemia were also repeatedly hospitalized for PRBC transfusions when they presented with symptomatic anemia. Children with hemophilia were cared for only when they presented with bleeding episodes. Although malnutrition was properly treated in the hospital, recurrent admissions of children with malnutrition were also noted. These situations highlighted a need to offer more effective preventive and supportive care outside the hospital and became the rationale for initiation of a comprehensive palliative care service.

The hospital already had a home care service in place for children affected by HIV due to the global effort in providing care to children with HIV to combat this enormous public health threat. This service was composed of experienced nurses and social workers and offered periodic home visits to assure compliance with antiretroviral therapy and to offer psychosocial and financial support. This team was occasionally assigned to provide home care visits for children with other conditions as well. All of the members of this team already had recognized the need and showed enthusiasm about extending their services to a wider population. This enthusiasm was the greatest strength of the hospital in formation of a comprehensive palliative care service for children.

#### Assessment of Need at the Hospital

An estimation of the need is a critical step in providing palliative care in children (16). Children with chronic, progressive, debilitating, or life-limiting illnesses are the real targets of palliative care. To identify the target population, we used previously published conditions that are considered to be appropriate targets and modified it to include other conditions frequently seen in Cambodia (17) (see Table 1).

**Table 1:** The conditions that could benefit from interdisciplinary palliative team approach.

*Conditions with potential cure*
Advanced or progressive cancer
Complex and severe congenital heart disease
Severe malnutrition
Life-threatening infections (sepsis, meningitis, etc.)
*Conditions requiring intensive long-term care*
HIV
Cystic fibrosis
Renal failure
Chronic severe respiratory failure
Muscular dystrophy
Chronic severe gastrointestinal disorders
Thalassemia major
Hemophilia
*Progressive conditions without cure*
Progressive metabolic disorders
Certain chromosomal abnormalities (trisomy 13, trisomy 18)
Severe form of osteogenesis imperfect
*Conditions involving severe non-progressive disability*
Severe cerebral palsy with difficult to control symptoms
Hypoxic anoxic brain injury
Extreme prematurity
Severe brain malformations

Next, the inpatient records of the first quarter of 2012 in AHC were retrospectively analyzed. Diagnostic coding information from the electronic database was utilized to identify patients with palliative care needs using the following scoring system: One point was given for chronic conditions, i.e., prematurity, asthma, kwashiorkor, marasmus, epilepsy, thalassemia, cerebral palsy, autoimmune disorders (excluding ITP), Down syndrome, hemophilia, and cardiac malformations; 1 point was given to those with life-threatening conditions, i.e., any ICU admission, any high mortality infection (septicemia, meningitis, HIV, neonatal infection, hemorrhagic Dengue fever); and 1 point was given for those without a possible curative therapy, i.e., most cancers.

During this time period, 668 patients were hospitalized 868 times with an average length of stay of 4.7 days. Thirty-six patients (5.4%) died in hospital during this period. Four hundred and three (60.3%) patients met at least one of the criteria for palliative care needs. When compared to those who did not meet any of the criteria, patients who met at least one criterion had longer hospital stays, higher readmission rates, and higher incidence of being hospitalized more than twice for the same reason. Nine patients (27% of those meeting the criteria for palliative care and needed repeated hospitalizations within 3 months) were hospitalized more than twice during this period. This information is summarized in Table 2.

**Table 2:** Comparison of characteristics of patients who are eligible for palliative care to those who are not based on the Angkor Hospital for Children 2012 first quarter survey.

	Do not meet criteria	Meets ≥1 criteria
Number of patients	265 (39.6%)	403 (60.3%)
Average length of stay (days)	3.3	5.6
Number of patients readmitted	7 (2.6%)	33 (8.2%)
Readmitted >2 times	None	9

Daily review of inpatient census during four consecutive weeks, we surveyed showed that at any given day approximately 60% of the inpatient beds were occupied by patients who would potentially benefit from palliative care based on the modified diagnostic criteria (see Table 1). This is in agreement with the results obtained using the point system on retrospective analysis of medical records.

#### Other Palliative Care Institutions in Siem Reap

There were no other palliative care or hospice organizations identified.

### Palliative Care Initiative at AHC

#### Introduction of a Modern Palliative Care Concept

The first phase of the formation of the team was an introduction to modern palliative care concepts to the hospital staff. For this purpose, the principal author had the opportunity to give two CME lectures on pediatric palliative care, one for all physicians, and another for all nurses. The WHO definition of palliative care is “an approach that improves the quality of life of patients and their families facing the problem associated with life-threatening illness, through the prevention and relief of suffering by means of early identification and impeccable assessment and treatment of pain and other problems, physical, psychosocial and spiritual” and was the definition utilized during these teaching sessions (18). We emphasized the fact that the modern palliative care begins when illness is diagnosed and continues regardless of whether or not a child receives treatment targeting the disease.

#### Formation of the Core Team

The second phase was the formation of the core team. For effective and sustainable palliative care, the team had to be consisted of experienced and motivated local providers. Through multidisciplinary meetings we identified the members of the hospital with the most experience in supportive care who are also highly motivated as core members of the palliative care team. A pediatric intensivist with additional training in pain management and an unmatched compassionate patient care was the best fit to lead this team. The remainder of the team consisted of a nurse from the HIV care team, a nurse from the home care team, and a social worker. The principal author acted as the consultant. The goals of the team were to provide inpatient and outpatient consultative services, home palliative care services, to coordinate transition between inpatient and outpatient care, to continually assess ways to improve palliative care both in the inpatient and the outpatient settings, to educate the hospital staff on an ongoing basis, and to explore research avenues to help improve care.

#### Intensive Palliative Care Training

The third phase of the process was training of the team members and other staff who showed interest in getting involved. Most presenters for the training sessions were selected among the motivated local providers in order to offer continuing education without the need of outside help. These included physicians, nurses, social workers, and medical educators. A full-day palliative care training workshop was held which was attended by 23 participants (including several participants from other non-governmental organizations in the area). The topics and speakers are presented in Table 3. An audience survey at the end of training session suggested that the workshop was considered effective with all of the speakers showing competence in their knowledge and ability to teach others. This training was repeated a year later during the principal author’s second visit. The second training session training was held in two half-day sessions and was repeated once (i.e., 4 half-day sessions in 2 days) to accommodate physicians’ and nurses’ schedule better. This led to an even a greater audience participation attesting to the success of these training sessions.

**Table 3:** Schedule of the first full-day workshop on pediatric palliative care at Angkor Hospital for Children.

Overview of palliative care (Mahmut Yaşar Çeliker)
Communicating with patients and families (Mahmut Yaşar Çeliker)
Management of pain and other physical symptoms (Yos Pagnarith)
Ethics, DNR, withdrawing, and withholding treatment (N. C. Pheaktra)
Questions and answers
LUNCH BREAK (with informal discussions)
Roundtable discussion on improving palliative care in IPD and ICU (all attendees)
Palliative care at home (D. Sophearin)
Responding to suffering and bereavement (Sokchea Sorn)
Questions and answers

#### Project Implementation

We initialized the formal activities of the team initially focusing on terminal and chronic severe conditions and ICU. The first service started was pain management, steroids, and home visits for the child with ALL mentioned previously. Inpatient consultation services were also initiated for numerous patients with life-threatening or chronic conditions. Those patients who were in most need of palliative care were given a phone number to contact with their questions and concerns (Yos Sophearin provided this phone number 24 h 7 days a week). Appropriate discharge planning was made for each patient with home visit plans. Periodic team meetings assessed the effectiveness of the team and identified gaps and other needs on an ongoing basis.

We carried out numerous home care visits with the nurse and social worker assessing patient status, identifying palliative care needs, and providing pain medications at home. In our index case, pain was adequately controlled, and the child started to walk and play again, and the family experienced decreased emotional suffering. We scheduled periodic outpatient visits to assess need for transfusion support in order to avoid emergency room visits. In a case where a child with metastatic liver cancer succumbed to his disease rather quickly, our team was effective in preparing the child and the family for end-of-life events and death at home allowing for a comfortable end-of-life transition. Our social worker provided bereavement visits following the death of the child. The feedback of the experiences from all the families was positive and encouraging. Follow-up interviews with the team members confirmed that palliative care inpatient and home services were continuing and that our index case was still living with a reasonable quality of life.

## Discussion

Sustainable and effective palliative care team must include adequately trained and motivated local health-care providers. Therefore, identification of appropriate members for such a multidisciplinary team is essential to success of such initiatives. In this regard, providers who are trained in supportive care measures within their own specialty (i.e., intensivist with additional training in pain management in this case), and recruitment of effective care providers from other focus groups (i.e., HIV home care team in this case) offer a great opportunity to develop integrated palliative care teams. Sustainability and effectiveness of this effort in our hospital was confirmed by periodic reports from the team members that effective palliative care services initially offered to oncology patients are now extended to patients with bleeding disorders as well.

Health-worker training has been recognized as a critical component in scaling-up of high-quality care and this was heavily researched in HIV and tuberculosis (19). The need for palliative care training in LMIC was highlighted in a recent review of published data (20). Formation of a multidisciplinary team in a resource-limited setting has many challenges ranging from correctly identifying the need to increasing resource availability and utilization (20, 21). Training of local providers by visiting faculty, although very effective, is not sufficiently empowering or sustainable unless local providers themselves are trained to become trainers in order to provide ongoing education periodically. In this regard, members of the local bioethics committee, medical educators, and well-trained physicians, nurses, and social workers as educators were effectively utilized in AHC and may form a model for other resource-limited settings.

Since resource allocation remains to be a challenge, recruitment of institutional support for activities of any interdisciplinary team requires a proper need assessment. We have utilized a point system and reviewed electronic medical records to quantitate the need for palliative care services in our hospital. The method has its shortcomings such as possible over representation in milder conditions and underrepresentation of patients who were not hospitalized. For example, thalassemia trait patients who do not have symptoms of anemia and are admitted for unrelated illnesses are not separated from transfusion-dependent thalassemia major patients. In contrast, patients whose parents refused hospitalization at time of diagnosis of severe illness were inadvertently left out. Since abandonment remains to be a major challenge in resource-limited settings, we believe this group may actually be larger than estimated and requires a separate investigation. Daily review of inpatient census during four consecutive weeks showed that at any given day approximately 60% of the inpatient beds are occupied by patients who would potentially benefit from palliative care based on the modified diagnostic criteria (see Table 1). This is in agreement with the results obtained using the point system and retrospective analysis of medical records. However, a prospective study to assess the validity of this method is needed. The data were presented at the appropriate steering committee meetings to highlight the importance of this activity. In institutions where an electronic database is not available, the tool presented here can be modified by prospectively evaluating cases in a predefined period of time to make an accurate assessment.

During our efforts to form an effective palliative care team, we have also identified obstacles to effective care in this setting. Below are the common themes for many settings of low and middle-income countries:

1. In a country afflicted with poverty, allocation of financial resources to benefit the general population takes priority. As a result, allocating sufficient resources to aid children with palliative care needs was a great challenge. Hospital administrators had to make financial decisions on a daily basis to distribute financial and human resources appropriately. Allocating sufficient resources for palliative care of a family was easily overcome by meeting the needs of a large number of other people with curable conditions. This is the greatest challenge Cambodia is facing and more international support is urgently needed.
2. Conditions that are amenable to palliative care are frequently considered to be “hopeless” by even the well-educated medical professionals. As a result, although there exist a great deal of empathy, motivation to do more, or supporting such initiatives is inadequate. We hope that this mentality will change with improved public health education and education of medical community.
3. Health-care professionals are working to full capacity in caring for acute and curable conditions. For proper execution of palliative care, more time per patient is required than is available. Additionally trained staff will be necessary to extend palliative care to all who needs it.
4. Social work, an essential component of a palliative care team is a relatively new concept to Cambodia’s medical community. The few social workers who are trained to handle a palliative care role are consumed by the enormous need of their services by an abundance of child abandonment and abuse cases in Cambodia. This high need in the community necessitates training of more social workers. Increased resource allocation is strongly linked to more training opportunities. Thus, support from the governmental and medical community is needed. In addition, child psychology is still a new concept to the Cambodian medical community but would have otherwise strengthened the palliative care team.
5. There is some degree of mistrust in modern medicinal approaches fueled by hundreds of years of traditions, low level of education, and competing interests of traditional healers. Traditional healers are frequently employed in the care of children from mild illnesses to cancer. As a result, both early diagnosis and appropriate care can be delayed, and in cases of terminal conditions, some families refuse modern medical care to seek help from traditional healers. In addition, the medical community, traditional healers, and religious figures are markedly polarized making collaboration very difficult. We hope that palliative care teams will gradually bring these polarized groups together to gain the trust of families and align their common goals to support the health of families.
6. Cambodia lacks an effective oversight system for medical practices. The care standards are set by the individual health-care centers rather than an overarching system. This leads to significant variability not only in the care provided but also in the type of care offered. AHC, which is managed with very high standards, is in a unique position to be a leader in developing such oversight committees with the support of governmental agencies.

## Conclusion

As a result of our experience in Cambodia, we make the following recommendations to be considered in forming effective multidisciplinary teams:

1. Allocation of resources (human and financial) requires proper assessment of the need. This may provide an effective tool for policy makers to understand the problem and consider appropriate action at the institutional as well as regional/national level. We recommend that every effort is made to obtain and properly analyze quantitative data.
2. Sustainable care requires motivated team of well-trained local providers. Reaching out to local hospitals and community resources for recruitment is a cornerstone of success in such initiatives.
3. Continuing education of palliative care providers requires ongoing training. We recommend training qualified local health-care providers, bioethicists, educators, and other relevant community members as trainers of palliative care.

Palliative care is increasingly being recognized as an essential part of care worldwide. It may be the only option available to some children. Effective palliative care can be offered in populations with limited resources by utilizing and building on existing resources. In addition, forming training teams of local experts assures continuing education of the providers. Our initiative at AHC is a proof of this concept. This team was formed with relatively low cost in a short period of time and was centered on sustainability. It could provide an invaluable model for training desperately needed expertise for other institutions in Cambodia and other resource-limited countries.

## Author Contributions

All authors (MÇ, YP, KA, DS, and SS) have met all four criteria for authorship.

## Conflict of Interest Statement

The authors declare that the research was conducted in the absence of any commercial or financial relationships that could be construed as a potential conflict of interest. The reviewer, RE, and the handling editor declared their shared affiliation, and the handling editor states that the process nevertheless met the standards of a fair and objective review.
